# Biofilm Development on Urinary Catheters Promotes the Appearance of Viable but Nonculturable Bacteria

**DOI:** 10.1128/mBio.03584-20

**Published:** 2021-03-23

**Authors:** Sandra A. Wilks, Verena V. Koerfer, Jacqui A. Prieto, Mandy Fader, C. William Keevil

**Affiliations:** a School of Health Sciences, University of Southampton, Southampton, United Kingdom; b School of Biological Sciences, University of Southampton, Southampton, United Kingdom; c University Duisburg—Essen, Essen, Germany; CEH-Oxford

**Keywords:** biofilms, silver, urinary catheters, viable but nonculturable

## Abstract

Catheter-associated urinary tract infections have serious consequences, for both patients and health care resources. Much work has been carried out to develop an antimicrobial catheter. Although such developments have shown promise under laboratory conditions, none have demonstrated a clear advantage in clinical trials. Using a range of microbiological and advanced microscopy techniques, a detailed laboratory study comparing biofilm development on silicone, hydrogel latex, and silver alloy-coated hydrogel latex catheters was carried out. Biofilm development by Escherichia coli, Pseudomonas aeruginosa, and Proteus mirabilis on three commercially available catheters was tracked over time. Samples were examined with episcopic differential interference contrast (EDIC) microscopy, culture analysis, and staining techniques to quantify viable but nonculturable (VBNC) bacteria. Both qualitative and quantitative assessments found biofilms to develop rapidly on all three materials. EDIC microscopy revealed the rough surface topography of the materials. Differences between culture counts and quantification of total and dead cells demonstrated the presence of VBNC populations, where bacteria retain viability but are not metabolically active. The use of nonculture-based techniques showed the development of widespread VBNC populations. These VBNC populations were more evident on silver alloy-coated hydrogel latex catheters, indicating a bacteriostatic effect at best. The laboratory tests reported here, which detect VBNC bacteria, allow more rigorous assessment of antimicrobial catheters, explaining why there is often minimal benefit to patients.

## Abstract (precis)

Several antimicrobial urinary catheter materials have been developed, but, although laboratory studies may show a benefit, none have significantly improved clinical outcomes. The use of poorly designed laboratory testing and lack of consideration of the impact of VBNC populations may be responsible.

## INTRODUCTION

Urinary tract infections (UTIs) are the second most frequent cause of health care-associated infections among hospitalized patients across Europe, with 60% attributable to indwelling urinary catheterization (catheter-associated UTI [CAUTI]). The use of a catheter increases the likelihood of bacteriuria (1, 2). Indeed, recent microbiome research in the healthy bladder (3, 4) has shown urine not to be sterile, with asymptomatic bacteriuria routinely found when advanced molecular sequencing is used. Bacteriuria and the presence of a catheter result in a high risk of biofilm development, where bacteria attach to the catheter material, forming complex communities and increasing antibiotic resistance. Biofilms are known to have a role in CAUTI development and in catheter blockage, commonly caused by the presence of urease-producing bacteria.

The role of biofilms has been considered in laboratory-based studies (5–11), and there has been considerable work to develop antimicrobial materials (12–15). While several have shown promise during laboratory testing, few have been assessed clinically. An exception is the use of silver alloy-coated/impregnated catheters which became common in clinical settings, with *in vitro* evidence indicating a reduction in the incidence of CAUTI (16–18). However, in a large-scale clinical trial involving approximately 7,000 patients, Pickard et al. (19) demonstrated no difference in the incidence of infection between standard uncoated catheters and silver-impregnated/coated catheters in patients undergoing short-term catheterization. The trial did, however, note a reduction in bacteriuria in patients using the silver catheters.

This raises important questions as to why the trial data differed from what laboratory studies predicted. It would be expected that a polymicrobial community and the presence of human tissue *in vivo* would affect activity compared to that under controlled laboratory conditions. Also, it is known that bacteriuria does not inevitably lead to infection. However, the analytical techniques used and metabolic state of the bacteria can also have an impact, and these are often neglected in studies.

To assess the antimicrobial activity of a material, attached cells are often removed and placed on nutritious agar media. This can lead to an underestimation of a biofilm population due to inefficiencies in removal and the presence of viable but nonculturable (VBNC) bacteria. VBNC bacteria arise from cells being sublethally stressed and being unable to grow on rich nutrient media, thus leading to an underestimation of population density (20–22). VBNC bacteria can retain infectivity (22, 23) and may be implicated in chronic recurring infections, as this metabolic state can be induced by the action of antibiotics (21, 24). Previously described methods to study biofilm development on urinary catheters can be limiting, as outlined recently (11). It is also important to consider that bacteria within a biofilm state can have an altered phenotype and behave differently than their planktonic counterparts.

In the present study, a combination of *in situ* advanced microscopy (11, 23) and *ex situ* viability staining and culture analyses have been used to track biofilm development by three commonly found and clinically important bacterial uropathogens (Escherichia coli, Pseudomonas aeruginosa, and Proteus mirabilis) on three catheter materials, silicone, hydrogel latex, and silver alloy-coated hydrogel latex, in a physiologically correct artificial urine medium. Using techniques specific to the detection of biofilms and VBNC bacteria, we illustrate how different catheter materials affect bacterial attachment and biofilm formation and can lead to increased VBNC populations.

## RESULTS

### Unused catheters.

**FIG 1:**
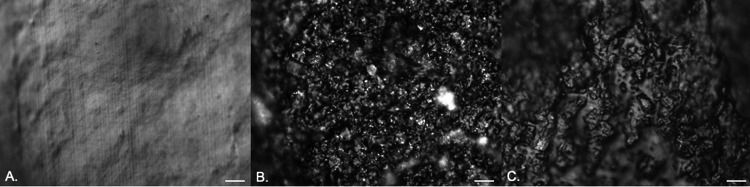
EDIC images of the surfaces of clean, unused catheters: silicone catheter (A), hydrogel latex catheter (B), and silver alloy-coated hydrogel latex catheter (C). Magnification, ×1,000; bars, 10 μm.

Episcopic differential interference contrast (EDIC) microscopy allowed rapid examination of silicone, hydrogel latex, and silver alloy-coated catheters (Fig. 1A to C). The silicone catheter (Fig. 1A) had a smooth topography, but there was evidence of pitting and undulations as well as parallel striations formed by extrusion during the manufacturing process. In contrast, the hydrogel and silver alloy-coated catheters (Fig. 1B and C) had highly disordered rough surface topographies. For all three materials, there were numerous potential bacterial attachment sites.

### Qualitative assessment of biofilm development.

**FIG 2:**
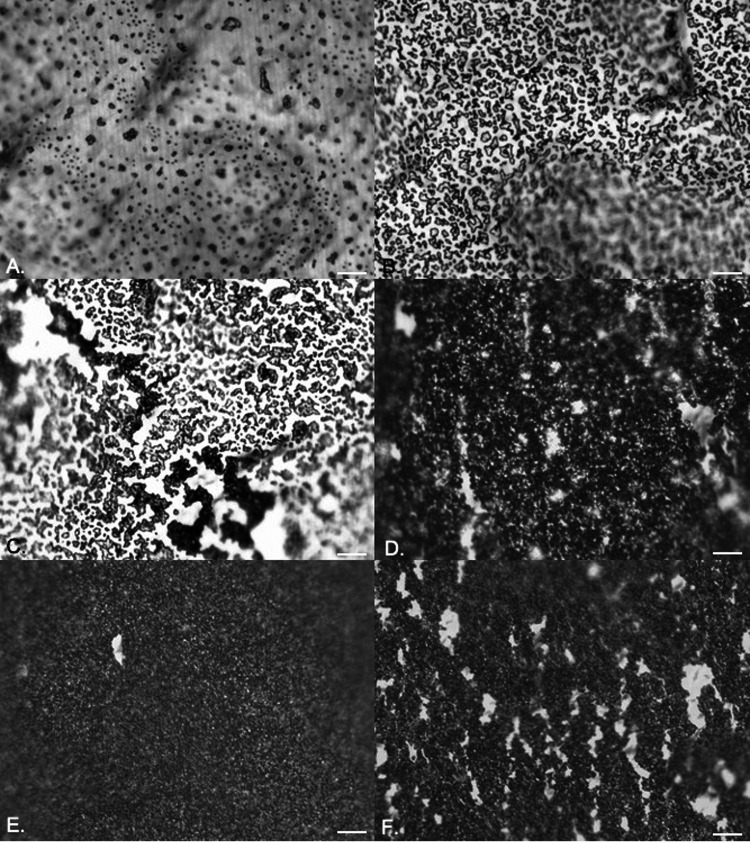
EDIC images showing attachment of E. coli to silicone catheters in artificial urine after 2 h (A), 4 h (B), 6 h (C), 24 h (D), 48 h (E), and 72 h (F) of exposure. Magnification, ×1,000; bars, 10 μm.

**(i) Silicone.** It was possible to use EDIC microscopy to visualize and track biofilm development on silicone catheters. Figure 2A to F shows an example sequence from initial attachment progressing to biofilm development when E. coli was inoculated into artificial urine. Over the first 6 h of exposure, there was a gradual increase in attached bacteria and material, from individual cells (2 h) to clear clustering and increasing density by 6 h (Fig. 2A to C). Following the 6-h exposure, distinct microcolony development was seen with associated extracellular polymeric substances (EPS) formation. After 24 h, a thick layer covered almost the entire surface, and this continued over 48 h (Fig. 2D and E). When sections were left for 72 h, channels and areas where biofilm had sloughed away were apparent, suggesting nutrient limitation, detachment events, and exposed areas ready for recolonization (Fig. 2F).

**FIG 3:**
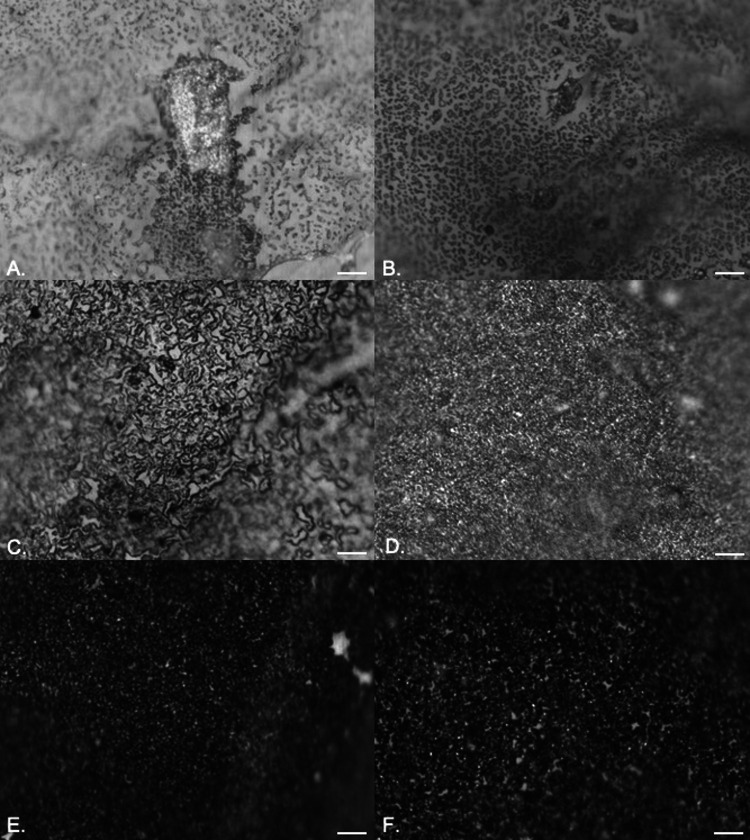
EDIC images showing attachment of P. aeruginosa to silicone catheters in artificial urine after 2 h (A), 4 h (B), 6 h (C), 24 h (D), 48 h (E), and 72 h (F) of exposure. Magnification, ×1,000; bars, 10 μm.

**FIG 4:**
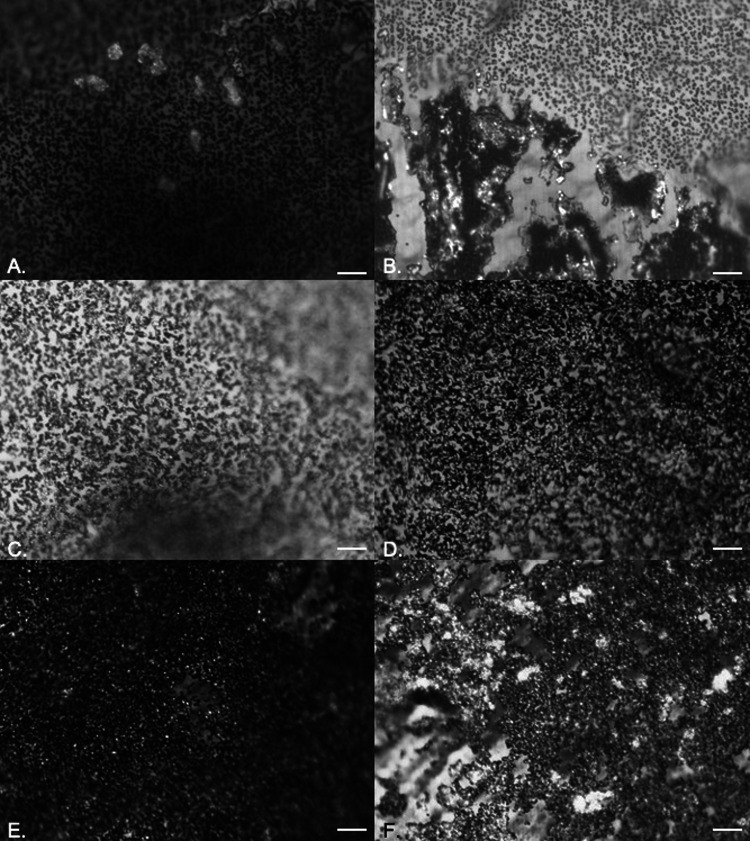
EDIC images showing attachment of P. mirabilis to silicone catheters in artificial urine after 2 h (A), 4 h (B), 6 h (C), 24 h (D), 48 h (E), and 72 h (F) of exposure. Magnification, ×1,000; bars, 10 μm.

Similar results were obtained for both P. aeruginosa (Fig. 3A to F) and P. mirabilis (Fig. 4A to F). P. aeruginosa rapidly colonized the surface, leading to increased microcolony formation and large amounts of extracellular polymeric substances (EPS). After 24 h of exposure, thickening of the biofilm was observed with stacks of microcolonies extending out from the surface (Fig. 3D). There was no evidence of sloughing or detachment at increased exposure times (Fig. 3E and F), but areas with increased reflectance indicated crystal formation and deposition due to urease activity. For P. mirabilis, single-cell attachment occurred rapidly, and after 6 h of exposure, an open mosaic structure was observed (Fig. 4C). As reported previously (11), this was followed by almost complete coverage of the surface, with evidence of crystal formation clear by 48 h, and after 72 h, large areas had sloughed away, creating sites for recolonization (Fig. 4D to F).

### (ii) Hydrogel latex/silver.

**FIG 5:**
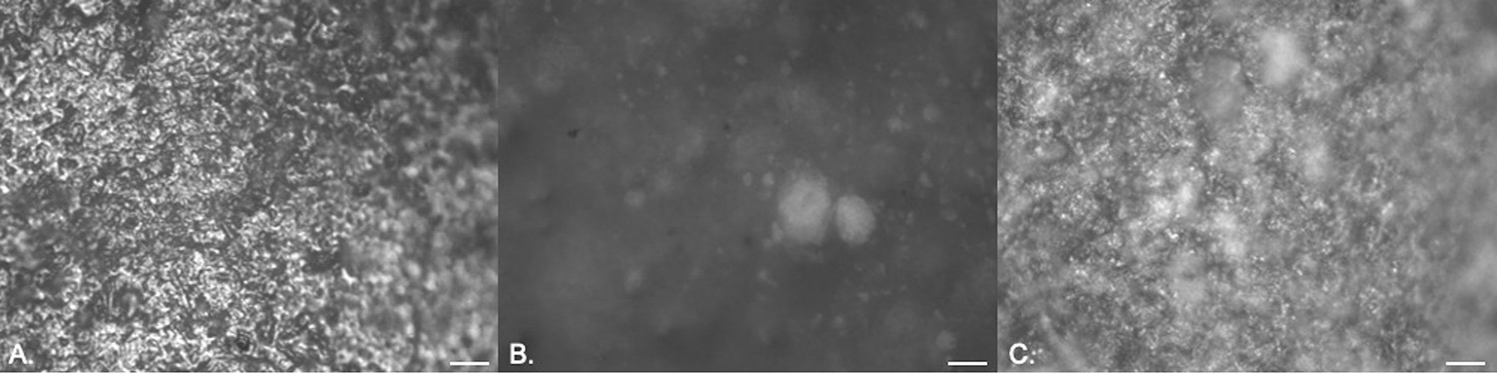
EDIC images showing attachment of P. aeruginosa to hydrogel latex catheters in artificial urine after 24 h (A), 48 h (B), and 72 h (C) of exposure. Magnification, ×1,000; bars, 10 μm.

**FIG 6:**
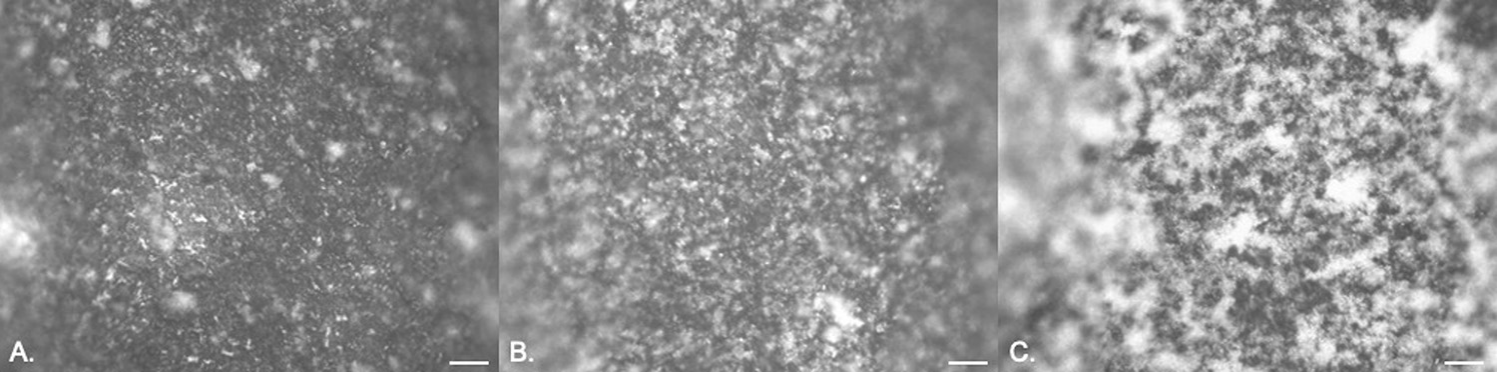
EDIC images showing attachment of P. mirabilis to silver alloy-coated hydrogel latex catheters in artificial urine after 24 h (A), 48 h (B), and 72 h (C) of exposure. Magnification, ×1,000; bars, 10 μm.

It was not possible to follow the early stages of bacterial attachment on either material due to the highly disordered surface topographies. Some evidence of crystalline biofilm development was seen over longer exposure times (24, 48, and 72 h of exposure) for P. aeruginosa (Fig. 5A to C) and P. mirabilis (Fig. 6A to C) due to urease production.

### Quantitative assessment of biofilm formation.

Three methods were used to quantify biofilm development: staining with SYTO 9 (total cell counts [TCC]), staining with propidium iodide (PI) (dead cells [dead]), and culture analysis (CFU counts).

### (i) E. coli.

**FIG 7:**
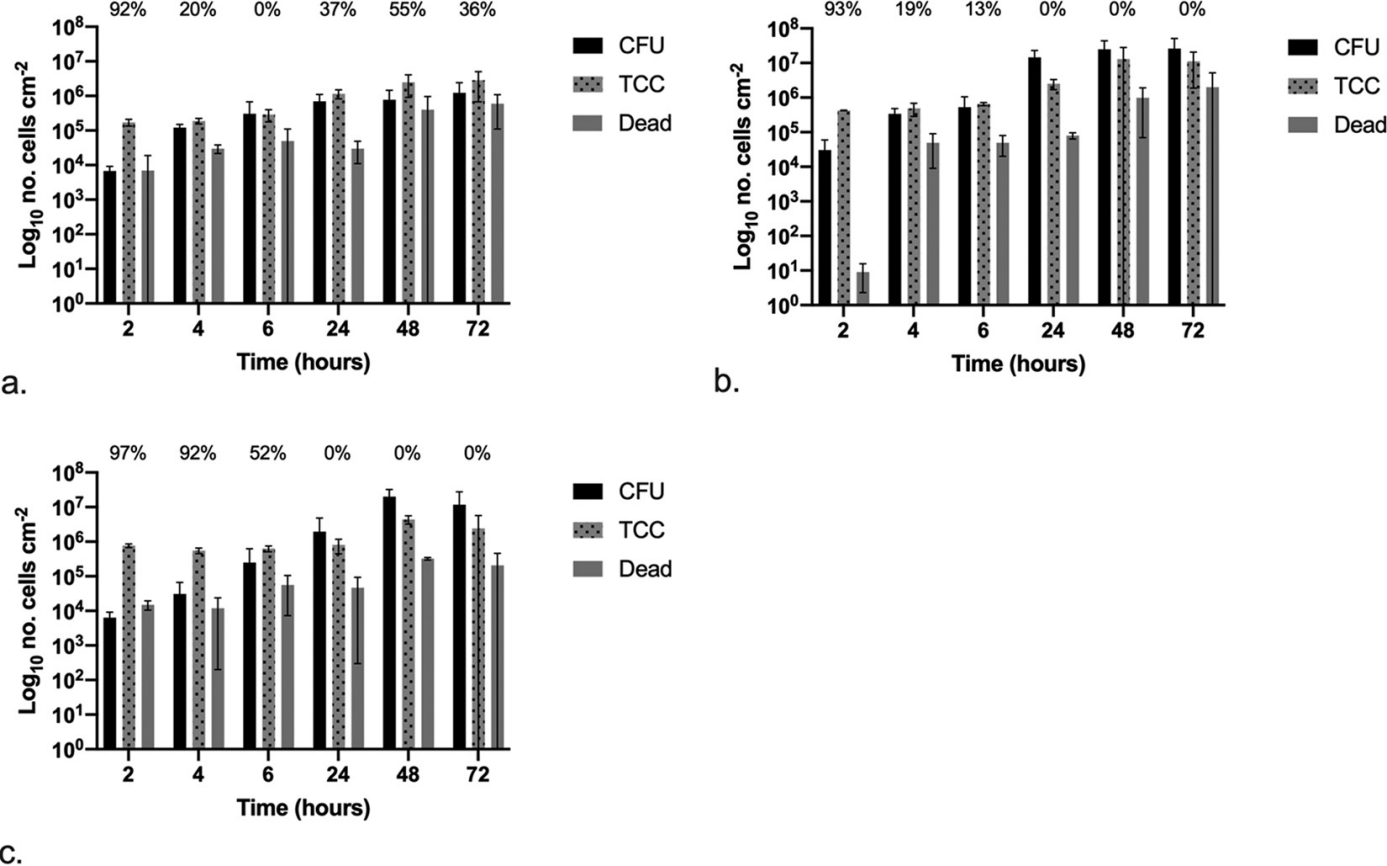
Graphs showing the numbers of CFU, total cell counts (TCC), and dead cell counts (dead) per centimeter squared over time following exposure to E. coli: silicone (a), hydrogel latex (b), and silver alloy hydrogel latex (c) catheters. The percentages of the VBNC population (CFU plus dead cell counts) in relation to total cell counts are shown.

The attachment and development of E. coli biofilms over 72 h was assessed and quantified, with results shown in Fig. 7a to c, for silicone, hydrogel latex, and silver alloy-coated hydrogel latex, respectively, with the percentage of the population in a VBNC state given (refer to the equation below). There were no significant differences between CFU, TCC, and dead counts over time or between catheter materials (*P* < 0.05).

$$\text{\% VBNC}=\frac{\left[\text{TCC}-\left(\text{CFU}+\text{dead}\right)\right]}{\text{TCC}}\times \text{1}00.$$

The equation is for the calculation of the percentage VBNC cells within the total population, where TCC is total number of cells (SYTO 9 labeled), CFU represents culturable cells, and dead is for the nonviable cells (PI labeled).

On silicone (Fig. 7a), after 2 h of exposure, there was a >1-log difference between the sum of CFU plus dead cells and the TCC counts, suggesting at least 90% of the population was VBNC. This was also observed after 48 and 72 h of exposure, where there was an approximately 0.5-log difference. On hydrogel latex (Fig. 7b), after 24 h and greater exposure times, the CFU counts were equal to or greater than the TCC, with many cells undergoing cell division when examined under the microscope. The early time points did show evidence of VBNC populations, but these decreased from 93% after 2 h to 13% at 6 h. A different pattern was found on silver alloy-coated hydrogel latex catheters (Fig. 7c); although CFU counts were ≥TCC at 24 h and longer exposure time points, in the first 4 h, the sum of CFU plus dead cells remained between 1 and 2 log lower than TCC, suggesting the presence of a VBNC population of >90%, only decreasing to 52% at 6 h.

### (ii) P. aeruginosa.

**FIG 8:**
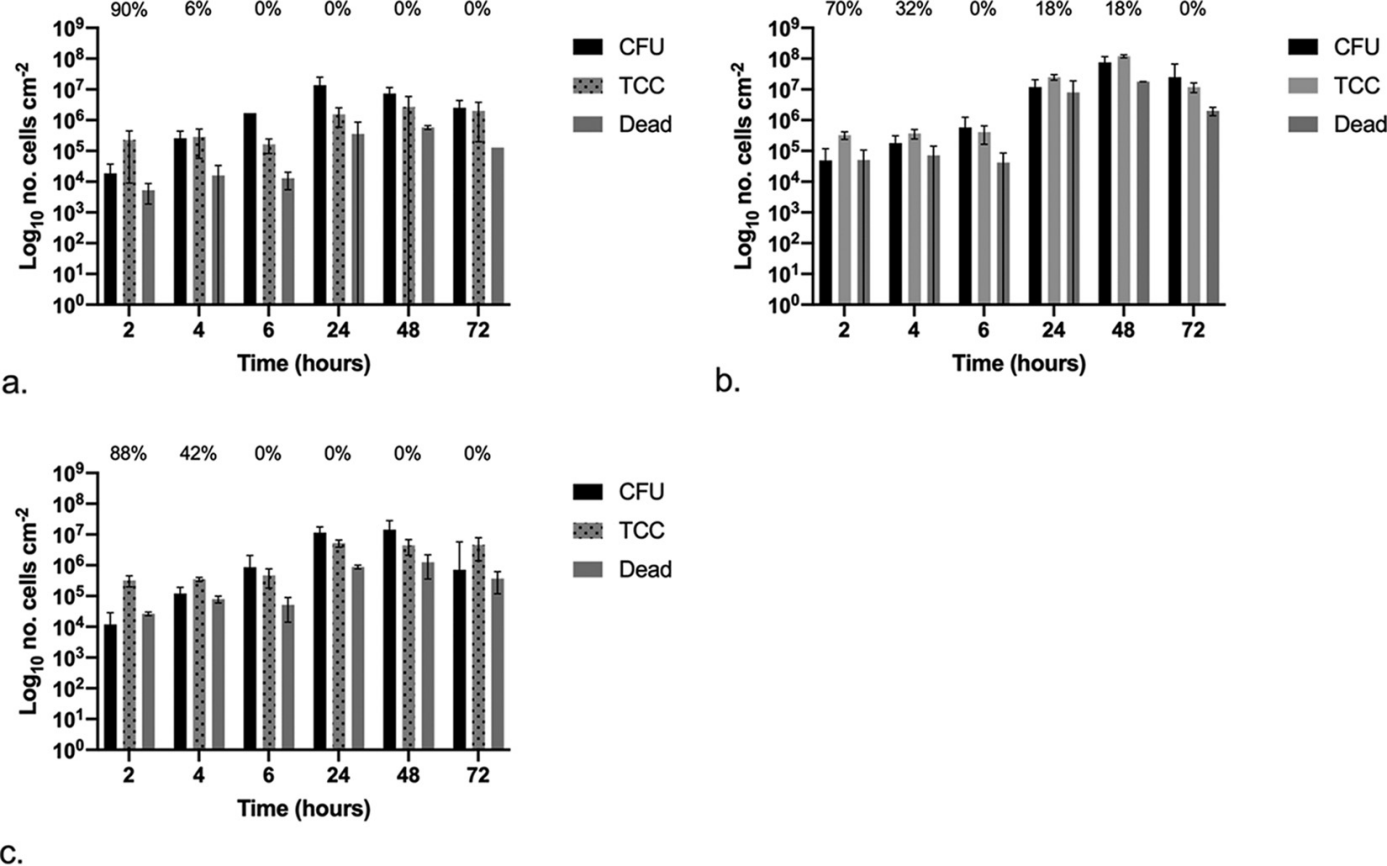
Graphs showing the numbers of CFU, total cell counts (TCC), and dead cell counts (dead) per centimeter squared over time following exposure to P. aeruginosa: silicone (a), hydrogel latex (b), and silver alloy hydrogel latex (c) catheters. The percentages of the VBNC population (CFU plus dead cell counts) in relation to total cell counts are shown.

The attachment and development of P. aeruginosa biofilms over 72 h was assessed and quantified, with results shown in Fig. 8a to c for silicone, hydrogel latex, and silver alloy-coated hydrogel latex, respectively, with the percentage of the population in a VBNC state given (refer to the equation above). When comparing the three materials, there were some significant differences (*P* < 0.05). Dead cell counts were significantly different at the longer exposure times (≥24 h), with counts for silicone less than those for silver alloy, which were less than those for hydrogel latex. However, at 48 h, TCC were also significantly different in the same order, with the highest values recorded for hydrogel latex. The only significant difference for CFU values was after 72 h, when results for hydrogel latex (Fig. 8b) were almost 2-log higher than those for silver alloy-coated hydrogel latex (Fig. 8c).

On silicone (Fig. 8a), at 2 h of exposure, CFU values plus dead counts were >1-log lower than TCC, indicating the presence of VBNC cells (90% of the population). However, at later time points, CFU counts were greater than TCC, with many cells in the process of dividing and hence underestimated, with no evidence of VBNC formation. For hydrogel latex (Fig. 8b), there was little difference between TCC and CFU, although 70% of the population was in a VBNC state at 2 h and 32% after 4 h. This pattern was followed but increased on silver alloy-coated hydrogel latex (Fig. 8c): CFU plus dead cells were approximately 1-log lower than TCC after 2 h, with 88% of the population in a VBNC state reducing to 42% at 4 h.

### (iii) P. mirabilis.

**FIG 9:**
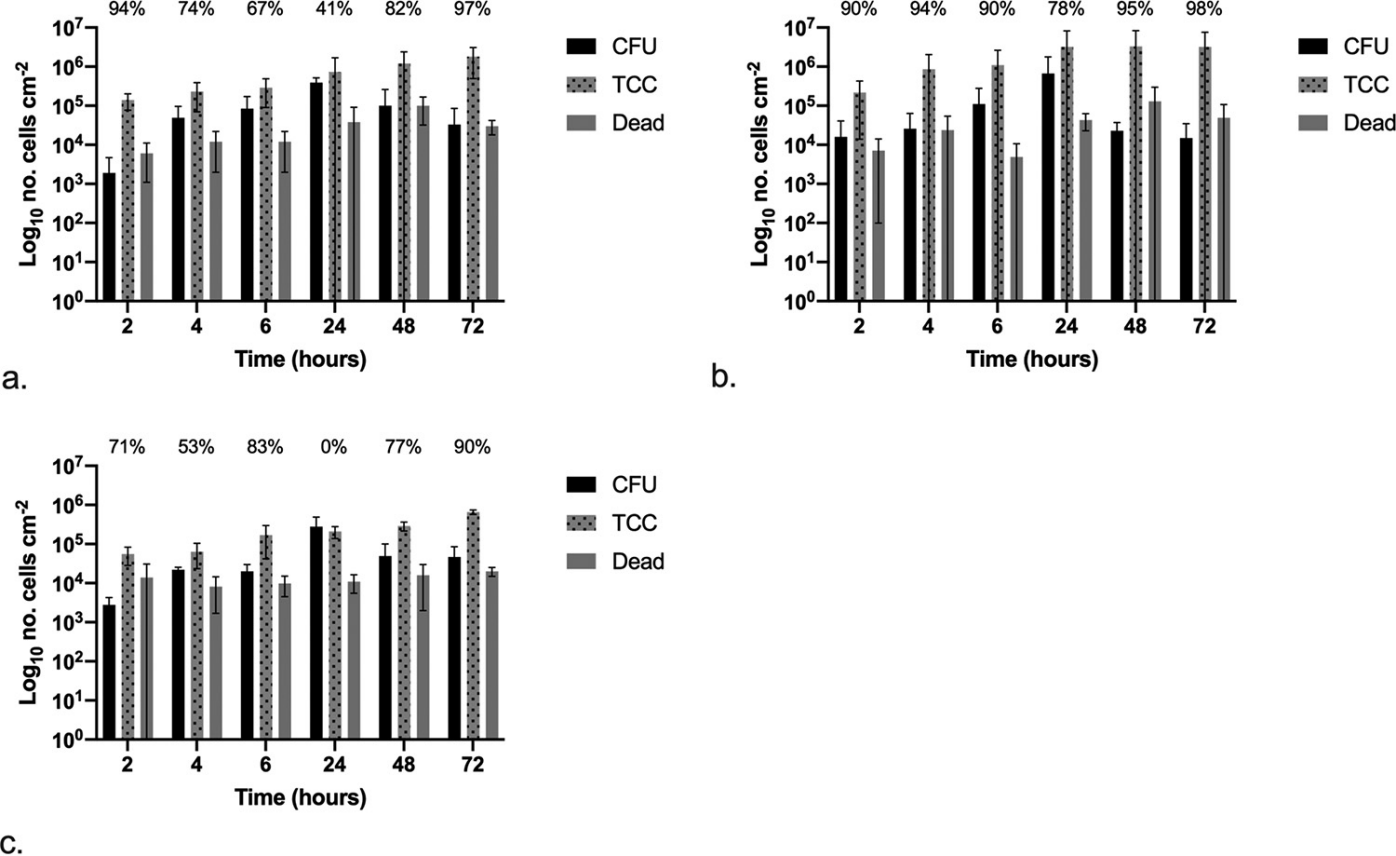
Graphs showing the numbers of CFU, total cell counts (TCC), and dead cell counts (dead) per centimeter squared over time following exposure to P. mirabilis: silicone (a), hydrogel latex (b), and silver alloy hydrogel latex (c) catheters. The percentages of VBNC the population (CFU plus dead cell counts) in relation to total cell counts are shown.

The attachment and development of P. mirabilis biofilms over 72 h was assessed and quantified, with results shown in Fig. 9a to c for silicone, hydrogel latex, and silver alloy-coated hydrogel latex, respectively, with the percentage of the population in a VBNC state given (refer to the equation above). When comparing materials, the TCC values were significantly different at all time points, with those for silver alloy lower than for silicone which were lower than those for hydrogel latex. The CFU and dead cell counts did not differ significantly across materials.

On silicone (Fig. 9a), there were no significant differences over time; however, there was evidence of VBNC populations at all time points, with an approximately 2-log difference between the sum of CFU plus dead cells and TCC after 2- and 72-h exposure times (94 and 97%, respectively). This was also seen on hydrogel latex (Fig. 9b), with an approximately 2-log (≤95%) difference at the longer exposure times of 48 and 72 h. Likewise, with the exception of 24 h, the number of CFU was always >1 log (ranging between 53 and 90%) lower than the TCC on silver alloy-coated hydrogel latex (Fig. 9c).

## DISCUSSION

Despite continued efforts to produce effective antimicrobial catheter materials and coatings that resist biofilm development, the problems of CAUTI and blockage prevail. To date, no material has been found to improve clinical outcomes significantly over long-term use, and many are described as being bacteriostatic rather than bactericidal.

The use of silver alloy coatings and impregnated catheters has been shown to reduce bacterial numbers in *in vitro* studies (16–18, 25). Indeed, Gabriel et al. (25) reported on the efficacy of a silver-coated catheter in 1996, leading to subsequent clinical approval. While silver-coated/impregnated catheters became widely adopted, several *in vivo* studies have indicated that any positive benefit of using silver-coated or -impregnated catheters is short term only (<7 days) (26–29), with a delay in colonization observed. In these cases, the antimicrobial action of silver can, at best, be described as bacteriostatic. This was also demonstrated in a large, randomized multicenter trial (19), where no reduction in CAUTI following the use of silver alloy-coated catheters compared to that with nitrofural-impregnated and polytetrafluoroethylene (PTFE)-coated catheters was found. Results from this study indicated that to prevent one incidence of CAUTI, at least 1,000 people would need to be using the silver alloy-coated catheter. This contradicts the findings of previous studies (30, 31), which had led to the recommendation of silver alloy-coated catheters for routine short-term use in the United Kingdom and the United States. This questions whether appropriate *in vitro* studies could have prevented the subsequent recommendation for clinical use. Indeed, early studies such as that by Johnson et al. (16) did report that alternative antimicrobial catheter materials, such as those containing nitrofurazone, significantly outperformed silver hydrogel catheters.

In the present study, we have tracked biofilm formation and investigated whether metabolically inactive VBNC populations arise on three catheter materials, including a silver alloy, and could be responsible for clinical outcome failures.

The VBNC state remains poorly understood, although it has been reported to occur in a wide range of bacterial species covering most phyla (21). The majority of work has focused on nonclinical environments, including water (32–34) and food production (22), with several referencing biofilms as providing a reservoir niche. The impact of VBNC bacteria on clinical infection risk remains poorly understood, with the role of persisters (cells that demonstrate antibiotic tolerance with restored growth on/in nutrient media once the stress is removed) more frequently studied (35). There remains debate on the similarities and differences on the metabolic state of persister and VBNC cells (21, 36, 37), with a continuum in metabolic activity from dead to actively growing seeming likely (21). Moreover, several studies (22, 34) have demonstrated how VBNC cells, even in a “deeper” level of dormancy, can lead to infection in animal models. Additionally, it is well known that any medical device which enters the body provides a high-risk interface for bacterial attachment and biofilm formation. It is also known that both VBNC and persister cells can be commonly isolated from biofilms where stressors such as nutrient depletion, redox gradients, and pH/ionic changes can occur.

Considering these factors and our recent understanding of the diverse urinary microbiome (3), the potential for VBNC populations to form on urinary catheters is high; however, this has not been explored previously. As CAUTI (particularly in chronic recurring infections and blockages) and rapid biofilm development on urinary catheters are well documented but no antimicrobial material (whether impregnated or coated) has been successful clinically in long-term patients, the appearance of VBNC populations may be an important factor. Indeed, the implications of VBNC populations in any device-related contamination and infection have not been widely considered or studied.

Using a combination of qualitative and quantitative methods, we tracked bacterial attachment, biofilm formation, and the appearance of VBNC populations over time on three different catheter materials: silicone, hydrogel latex, and silver alloy-impregnated hydrogel latex. The use of EDIC microscopy (11, 38) demonstrated how, for all three species of bacteria tested, initial attachment was rapid, occurring in less than 2 h. E. coli showed biofilm maturation over the first 48 h before exhibiting a typical mosaic pattern with detachment events. In contrast, the biofilm-forming P. aeruginosa showed no signs of detachment, forming an extensive and thick biofilm with evidence of stack formation. The urease-producing species (P. mirabilis and P. aeruginosa) showed evidence of microcrystalline formation and, in the former, development of complex crystalline encrustations, which lead to catheter blockages. This corresponds to work by Wilks et al. (11), in which four distinct stages were identified.

Such qualitative observations are useful in understanding the susceptibility of catheter materials for biofilm development and gross structural characteristics but do not reflect differences in bacterial numbers or variations in viability and metabolic state. By using three quantitative methods, namely, culture analysis (culturable cell count) and separate enumeration of SYTO 9-labeled (total cell count) and PI-labeled (dead cell count) bacteria, we have shown the presence of an increasing VBNC population on silver alloy-coated catheters. If no VBNC population is present, culture data plus the numbers of dead cells (PI labeled) should equal the total cell count (SYTO 9 labeled). While results for P. aeruginosa did follow this pattern on all materials (other than at short time points), indicative of its behavior as a strong biofilm-forming species, uropathogenic E. coli and P. mirabilis did not. This implies that VBNC cells are a natural component of the urinary catheter-related biofilms for these two bacteria. However, increased numbers of VBNC cells were observed on the silver alloy-coated catheters and with little to no evidence of antimicrobial killing from this material type. Interestingly, a study by Zandri et al. (39) detected a VBNC population of Staphylococcus aureus in biofilms on central venous catheters removed from patients after implantation times of 3 days to 3 months (77% of 44 samples showed the presence of VBNC S. aureus).

The question arises why previous *in vitro* studies showed significant antimicrobial properties of silver alloy-coated/impregnated catheters, which led to rapid clinical adoption. Consideration must be given to how experiments were designed and the measurements collected. These studies have relied on the growth of bacteria in nutritious laboratory media (where VBNC populations will be missed) or in minimal media, neither accurately reflecting the physiological environment of the urinary system. Studies have used radiolabeled leucine (17, 25) to track bacterial adherence and agar diffusion, where inhibition of growth on sections of catheter materials was measured (16, 17) on Mueller-Hinton agar plates. Samuel and Guggenbichler (18) described four methods, including growth of bacteria released from catheter surfaces by turbidity readings, measurement of antimicrobial activity by the Dow shaker method (immersion in inoculated saline, and aliquots of the suspension plated after set amounts of time), and the roll plate technique (inoculated catheter sections are rolled over agar plates). The present study utilizes a physiologically correct artificial urine medium, which in itself influences the attachment and development of biofilms, including the impact of urease release and crystal formation (11). It then becomes clear how the mismatch between *in vitro* study design could have impacted the discrepancies seen with *in vivo* use.

Although the clinical significance of VBNC bacteria is not fully understood, there is increasing evidence that they may have an important role in chronic and persistent infections (20). The possibility of metabolically inactive bacteria having a role in UTI persistence was described by Mulvey et al. (40) who showed how a reservoir of inactive E. coli could be found inside bladder epithelial cells. The implications of such a population have not been explored further in relation to catheter-associated biofilm risk. Studies are demonstrating widespread retention of infectivity (22, 34), indicating that the VBNC state could be a key mechanism and that their increased antibiotic tolerance impacts the efficacy of treatment plans. Indeed, the presence of a VBNC population, as demonstrated here, is a possible explanation for why silver alloy-coated or -impregnated catheters have failed in clinical trials.

If these VBNC cells retain infectivity, this may account for why there was no reduction in CAUTI in the study by Pickard et al. (19), despite previous *in vivo* studies reporting a reduction in bacteriuria as found using standard urinalysis.

This work demonstrates the needs for rigorous testing of medical device materials prior to clinical trial and market release and for full understanding of the implications of bacteriostatic versus bactericidal populations, particularly considering the presence of VBNC populations. By combining several analysis methods, robust data can be obtained on the real antimicrobial activity of materials. This is vitally important in order to better understand the potential of new materials to reduce infection and blockage, improve patient care and quality of life, and reduce the financial burden of CAUTI.

## MATERIALS AND METHODS

### Bacterial inocula.

Escherichia coli NCTC 9001, Pseudomonas aeruginosa PAO1, and Proteus mirabilis NCTC 10975 were grown overnight at 37°C in tryptone soya broth (TSB) (Oxoid, UK). Aliquots were centrifuged at 3,780 × *g* for 10 min, and pellets resuspended in artificial urine medium (41).

### Biofilm development on catheters.

Three indwelling Foley catheters were tested: a 100% silicone (Rüsch), a hydrogel latex (Bard Biocath hydrogel), and a silver alloy-coated hydrogel latex (Bard Bardex I.C.). These were cut longitudinally and transversely to give approximately 1-cm^2^ surface area sections.

Catheter sections were placed in six-well tissue culture plates (two per well) and covered with 3 ml artificial urine (41). To test wells, the inoculum was added (to give a final concentration of approximately 10^8^ CFU/ml), with each well representing a separate time point. Control samples did not have bacterial inocula added. These plates were incubated at 37°C. Samples were taken after 2, 4, 6, 24, 48, and 72 h of exposure. Following removal from inoculated urine, each catheter section was gently washed with phosphate-buffered saline (PBS).

### Qualitative assessment of biofilm development.

One section was set aside for direct analysis by episcopic differential interference contrast (EDIC) microscopy using a Nikon Eclipse LV100D microscope with EXFO X-Cite 120 metal halide fluorescence system and long-working-distance metallurgical Nikon Plan Achromat objectives (Best Scientific, UK) (11, 38).

### Quantitative assessment of biofilm development.

The second catheter section from each well was used for indirect biofilm analysis. Biofilm was removed by scraping the surface with a sterile 1-μl inoculation loop, which was transferred to 5 ml of PBS and vortex mixed for 30 s. This resuspended biofilm was serially diluted in PBS, plated onto tryptone soya agar (TSA) (Oxoid, UK), and incubated at 37°C overnight. Resuspended biofilm samples were also stained with the SYTO 9/propidium iodide (PI) LIVE/DEAD BacLight system (Invitrogen, UK) to give total cell counts and numbers of dead cells. In each case, 1.5 μl of either SYTO 9 or propidium iodide (PI) was added to a 1-ml sample and incubated, in the dark, for 20 min. Following this, samples were filtered onto black 0.2-μm-pore-size polycarbonate filters (Whatman, UK) and placed onto glass slides. Filters were examined, under epifluorescence illumination, using oil immersion, and numbers of stained bacteria were counted across a random selection of 10 fields of view.

### Statistical analysis.

All experiments were repeated in triplicates. Results obtained for culturable bacteria, total, and dead cell counts were log transformed. Differences between analysis methods and catheter materials were assessed using a one-way analysis of variance (ANOVA) followed by Tukey’s multiple-comparison test (Prism, GraphPad Software Inc.). Differences were considered significant if the *P* value was <0.05.
